# Canola Meal in Poultry Diet: Impact on pH, Color, Drip Loss, Nutritional Composition and Oxidative Status of Fresh and Stored Meat

**DOI:** 10.3390/ani16091297

**Published:** 2026-04-23

**Authors:** Marta del Puerto, María Cristina Cabrera, Ayrton da Silva, Roberto Olivero, Alejandra Terevinto, Ali Saadoun

**Affiliations:** 1 Laboratorio Nutrición y Calidad de Alimentos y Productos, Departamento Producción Animal & Pasturas, Facultad de Agronomía, Universidad de la República (Udelar), Montevideo 11400, Uruguay; adasilva@fagro.edu.uy (A.d.S.); rolivero@fagro.edu.uy (R.O.); aterevinto@fagro.edu.uy (A.T.); asaadoun@fcien.edu.uy (A.S.); 2 Sección Fisiología & Nutrición, Facultad de Ciencias, Universidad de la República (Udelar), Montevideo 11400, Uruguay

**Keywords:** canola meal, broiler, chicken meat, polyphenols, lipid and protein oxidation

## Abstract

Canola meal, with environmentally friendly attributes, lower cost, and previous studies, is an interesting proteic source to partially replace the soybean meal for poultry nutrition. For that, in this work we aimed to investigate canola meal as a partial replacement of soybean meal in finishing poultry diets (21 to 49 days) on the productive performance, also including the impact on the quality, nutritional attributes and antioxidative status of valuable cuts of meat. Ninety-six 21-day-old chickens were assigned to four experimental diets (24/diet), with increasing doses of canola meal (CM 0, 2.5, 5 and 10%). Daily consumption, weekly live weight and post mortem carcass weight and yield were determined. At 24 h post mortem, pH, color (CIE L, a*, b*) and drip loss were measured in the breast, drumstick and thigh cuts. Fatty acid composition and health lipid indexes were also determined in the fresh cuts. The oxidative status of lipids and proteins, polyphenol and flavonoids content in fresh and in stored (7 days-display at 4–6 °C) in vacuum packaged cuts were determined. Including CM, up to 10%, the feed intake and growth of birds was not affected (*p* = 0.74 and *p* = 0.87 respectively). In meat, CM significantly decreased the drip loss (*p* < 0.05), the pH in breast and thigh (*p* = 0.01 and *p* = 0.05 respectively), a lower L and b in thigh and increased PUFAs in more oxidative cuts, with a strong interaction between dose and muscle type. There was no effect on lipid oxidation while carbonyls decrease at a 2.5% dose in fresh and stored cuts but there is an increase with higher ones. Flavonoids raise the maximum deposition in meat at 5% CM. In conclusion, CM can be included in finishing poultry diets, but high doses must to be adequately managed if performance and quality of meat criteria are considered together.

## Simple Summary

Canola meal is an attractive proteic source alternative to soybean meal with environmental and friendly attributes for poultry nutrition. In this study, increasing levels of canola meal (0, 2.5, 5 and 10%) were included in finishing poultry diets (21 to 49 days), and the productive performance, technological and nutritional attributes in valuable cuts, breast, drumstick and thigh cuts, were studied. Ninety-six chickens received soybean meal or canola meal, and feed intake, live weight, carcass weight, 24 h pH, color (L*: lightness, a*: redness and b*: yellowness), drip loss, fatty acid composition and health lipid indexes were measured. The oxidative status of lipids and proteins, polyphenol and flavonoids content in fresh and in stored (7 days-display) in vacuum package cuts were assessed. With inclusion up to 10% of canola meal, growth and feed intake were not affected. Also, drip loss and pH in breast and thigh cuts were decreased, and thighs had less L and b. Lipid oxidation was not affected in spite of the increase in PUFAs, but an increase in protein carbonyls was observed. However, lower levels of canola meal, 2.5 and 5%, were overall beneficial, likely due to flavonoids. All attributes were largely dependent on muscle type.

## 1. Introduction

The constant development of the food and energy industry has left a large number of by-products of affordable cost and high availability that are very promising for use as components in production animal diets [1]. Among them, it is worth highlighting canola meal, a by-product of the industry for obtaining edible oil, with a constant increasing production and a relatively lower cost than other protein sources of traditional use. Indeed, canola meal is cost-competitive with soybean meal, but also from an environmental perspective, local canola has better adaptation to water restriction, reduces greenhouse gas emissions, and supports pollinators, which is crucial for countries whose economy is based on agricultural products [1]. The nutritional contributions of rapeseed meal average values of 36 to 38% CP, adequate levels of sulfured amino acids, 10 to 12% CF, and 1.2% EE, depending on the process and degree of oil extraction [2,3]. Although its use in feeding broiler chickens has spread widely, there is still controversy about the level of inclusion in diets, and its effect on their performance, primarily to its content of antinutrients, such as glucosinolates, phytic acid, polyphenols, and fiber that could negatively affect the use of metabolizable energy [4,5]. In an attempt to avoid the negative effect due to fiber, studies conducted by Elbaz et al. [6] demonstrated that the substitution of 20% of soybean meal with a fermented canola meal with exogenous enzymes improved growth performance, nutrient digestibility, immunity, antioxidant capacity, and gut health. However, Elbaz et al. [6] conclude that it was necessary to add exogenous enzymes to obtain the improved nutritional quality of a high level of canola meal as 20%. Indeed, previous studies by Kocher et al. [7] in broiler chickens fed high inclusion levels of canola meal (>25%) reported reduced feed digestibility and growth performance. The authors associated these negative outcomes with a high content of dietary fiber and also, probably to other antinutritional factors such as glucosinolates, erucic acid, sinapine, and tannins. Reports from 2012 [8] and actual research in 2025 [9] conducted with high levels of canola meal, as measured by growth performance and feed efficiency in broiler chickens, indicated that the diet with canola meal does not produce good results in growth, weight gain, or feed conversion efficiency. Considering the attributes of canola cultivation, from an environmental point of view, are being better adapted to dryness and less water, fertilizer and pesticides requirements, the oil extraction as a valuable product, the high level of protein of the subproduct, canola meal, and its lower cost, it is strategic to study the potential of replacing soybean meal in poultry diet to contribute with the economy of production. In consequence, it is in this framework that we consider the study of the effects of replacing soybean meal with increasing moderate levels of canola meal on the productive performance and also by including the effect on the meat quality, the nutritional composition and the oxidative status of meat, fresh and stored for 7 days.

## 2. Materials and Methods

### 2.1. Animals, Housing and Diets

For this trial, canola meal was provided by ALUR (Alcoholes Del Uruguay, ANCAP, Capurro, 11700, Montevideo, Uruguay), coming from canola (Brassica napus sp oleifera) cultivated in the southwest region of the country. Canola meal is obtained as a by-product after extracting oils for human consumption using the solvent extraction method. The chemical composition of canola meal used in this work was analyzed at the Nutrition Laboratory of the Department of Animal Production and Pastures (Faculty of Agronomy, Udelar) following the AOAC methods [10]. The composition was as follows: dry matter (925.10), 89.90%; in a dry matter basis the content of crude protein (981.10, N Kjeldahl) was 37.27%; with a high protein digestibility in vitro [11], 88.99%; ashes (920.153), 5.21%; ether extract (991.36 Soxhlet extraction with hexane as solvent), 1.43%;crude fiber (978.10), 14.12%; ADF (973.18), 20.94%; Lignin (973.18), 10.44%; amylaseNDF (2002.04), 35.61%; dietary fiber (985.29), 39.12%. Also, fatty acids composition was determined: C14:0, 0.16%; C16:0, 8.49%; C16:1, 2.27%; C18:0, 1.08%; C18:1, 57.39%; C18:2n6, 24.6%; C18:3n3, 4.1% and erucic acid (C22:1), 0.31% of total fatty acids (following the procedure in da Silva et al.) [12]. The content of glucosinolates, 4.5 µmol/g [13] and sulforaphane, 0.045 µmol/g [14] was also measured. The total polyphenols contained was 7.34 g of Gallic Acid Equivalent/kg [15]. Additionally, lysine and methionine–cysteine were analyzed by an external laboratory following the AOAC method (994.12) with HPLC (Agilent 1260, Agilent Technologye, Santa Clara, CA, USA) coupled with pre- or post-column derivatization following acid hydrolysis with previous oxidation with performic acid for methionine showing values of 1.81% and 1.30% respectively. For trial with the birds, ninety-six male Cobb500^TM^ chickens, 21 days old, selected for health and weight homogeneity, were housed in groups of three animals, in wooden pens (0.90 m × 0.90 m) with rice husk bedding, individual feeder and waterer. They were maintained with photoperiods of 23 h light in a climate room. The conditions, temperature and environment, were homogeneous for each pen. Each bird was in a completely randomized design distributed into 4 treatments with 24 birds each one (8 pens with 3 birds/pens each diet) according to the diets detailed below. A control diet based on corn and soybean meal (0%; 21.3% CP, 3191 kcal/kg ME, 0.90% total Ca, 0.4% available P and 1.3% and 0.86% for lysine and methionine-cistine respectively) and three experimental diets with increasing levels of inclusion of canola meal in replacement of soybean meal (2.5%, 5% and 10% of total diet). The maximum level of inclusion for canola meal (10%) follows the directives of the European Commission [16] based on the glucosinolates level measured in the canola meal studied here and previous findings by Toth et al. [17]. The diets showed in Table 1 were formulated isoenergetic and isoprotein, based on the analytical values of major ingredients (corn, soybean, meat and bone meal) following the Cobbs500^TM^Guide (https://www.cobbgenetics.com/assets/Cobb-Files/2022-Cobb500-Broiler-Performance-Nutrition-Supplement.pdf accessed on 19 October 2023). Proximate analysis of experimental diets was carried out according to AOAC [10] methods as detailed above for dry matter, crude protein, crude fiber, ashes and ether extract. For gross energy and total calcium of experimental diets, we followed Pond et al. [18] and 985.35 of AOAC respectively, and it was carried out in the Food and Product Quality Laboratory of the Faculty of Agronomy, Udelar, Uruguay. Available phosphorus and digestible amino acids, lysine, methionine, arginine and threonine were calculated according to the Tablas brasileñas para aves y cerdos [19].

At 42 days, all the animals were slaughtered after fasting for 12 h [20] in a commercially authorized slaughterhouse by MGAP (Uruguay). Experimental trial, management, transport and sacrifice were in accordance with the good animal welfare practices approved by the Honorary Committee for Animal Experimentation, Udelar (CHEA, protocol N°702).

### 2.2. In Vivo Determinations: Growth and Feed Intake

During the experimental period, records were taken on the 24 birds by diet, daily and accumulated individual consumption, and weekly individual live weight. From this record, the weight gain per animal in the period and the conversion efficiency rate were calculated.

Individual weight was determined at the beginning (initial, 21 days), weekly and at the end of the protocol (final, 42 days). Weight gain in the experimental period was calculated by the difference between initial and final weight.

Conversion efficiency was determined as the ratio between the food consumed and the variation in live weight individually, in the experimental period (kg of food consumed/kg of weight gain).

### 2.3. Preparation of Samples Fresh and Stored

After sacrifice, carcasses were chilled at +4 °C for 24 h and transported to the laboratory and immediately the pH, color and *drip loss* were assessed as explained in the next section. Also, immediately, samples of right *Pectoralis*, *Ileotibialis* L. and *Gastrocnemius* muscles corresponding to breast, thigh and drumstick were withdrawn from each carcass and stored in vacuum-sealed polyethylene bags in a freezer at −20 °C (fresh samples) until being analyzed for fatty acids, oxidative status and polyphenols and flavonoids content. The left *Pectoralis*, *Ileotibialis* L. and *Gastrocnemius* muscles were packed in polyethylene bags, vacuum-sealed, in a refrigerated display case (4–6 °C) for 7 days (stored sample) and then in a freezer at −20 °C until being analyzed for polyphenols and flavonoids content and oxidative status.

### 2.4. Meat Quality: pH, Color and Drip Loss

At 24 h post mortem, meat quality studies were carried out on the fresh right and left cuts from the breast (mainly m. *Pectoralis*), thigh (*Ileotibialis lateralis* m.) and drumstick (*Gastrocnemius* m.) of 24 birds by diet/cut, following Rose et al. [21]. The pH in each cut was determined with an LT Lutron pH-201 penetration pH meter, with three measurements by cut. The color was determined according to the CIELab method (L*: lightness, a*: redness and b*: yellowness; CIE, 1976) with a Konica Minolta Lab CR-10 Tristimulus Colorimeter, with standard D65 illuminant. Drip loss was determined according to the method of Penny [22] with slight modifications by da Silva et al. [12]. Samples of 5 g of each cut were taken, weighed and suspended in closed polyethylene bags, in a refrigerated environment at 4 °C. After 24 h, they were weighed again. The drip loss was determined as the water loss in % of weight differences at both times.

### 2.5. Fatty Acid Profile

Intramuscular lipid extraction was carried out following Folch et al.’s [23] procedure with slight modifications, as in da Silva et al. [12]. A sample of 2 g of each cut (with mainly *Gastrocnemius*, *Iliotibialis lateralis* and *Pectoralis* muscles, with visible fat removed) was homogenized at 12,000 rpm with an IKA T25 Homogenizer (IKA-Werke GmbH & Co. KG, Staufen, Germany), during 1 min, with 50 mL of chloroform/methanol (2:1). After that, the homogenate was filtered on fritted funnel (Graduation M), moved to a separating funnel, mixed by inversion for 1 min, and decanted overnight. The lower phase (chloroform containing lipids) was recuperated in a glass balloon, evaporated at 45 °C with a light vacuum in a Rotavapor (IKAbasic, IKA-Werke GmbH & Co. KG, Staufen, Germany). Afterward, the balloon was dried in an oven at 35–40 °C for 30 min and cooled at ambient temperature overnight in a vacuum desiccator. To determine the percentage of lipids of each sample the balloon was weighted at 0.0001 g. The methylation of fatty acids followed the procedure described by Ichihara et al. [24], using methanolic KOH. The determination of fatty acids by gas chromatography followed the procedure according to Eder [25], using fused-silica capillary column CPSIL-88 (Agilent, Santa Clara, CT, USA) of 100 m installed in a split/splitless chromatograph Clarus 500, and the samples (1 µL in hexane) were injected using an autosampler (Perkin Elmer Instruments, Shelton, CT, USA).

### 2.6. Calculus of Nutritional and Lipids Health Indices

Selected indices were calculated to establish the nutritional characteristics of meat chickens fed diet canola meal as partial substitution of soybean meal. Those indices were total SAT, MUFA and PUFA, total n-6 fatty acids, total n-3 fatty acids, n6-n3 ratio, and P/S ratio [12]. Furthermore, the usual lipids health indices were also calculated as follows:

Atherogenicity Index (AI): determined according to the formula proposed by Ulbricht and Sauthgate [26], using the ratio between pro-atherogenic fatty acids (SFA) and anti-atherogenic fatty acids (PUFA).

Thrombogenesis Index (TI): determined by the ratio between pro-thrombogenic fatty acids (SFA) and anti-thrombogenic fatty acids (MUFA + PUFA) according to the formula of Ulbricht and Sauthgate [26].

Hypocholesterolemic/Hypercholesterolemic ratio (h/H): According to the formula of Fernandez et al. [27], it is defined as the ratio between unsaturated fatty acids (MUFA and PUFA) and saturated fatty acids C14:0 and C16:0.

### 2.7. Total Polyphenol and Flavonoids Content

In 12 fresh and 12 stored samples of each cut/diet, total polyphenols and flavonoids were determined. Total polyphenols were determined according to Singleton et al.’s [15] method. Briefly, 0.8 g of meat in 20 mL of deionized water was homogenized at 8000 rpm for 20 s (Ultraturrax, T18 with S18 pointer, IKA, Staufen, Germany) and then centrifuged at 2000× *g* for 10 min, then filtered using Whatman 1 filter paper (Merck, Darmstadt, Germany. One hundred µl of the filtrate were vortexed with 150 µL of Folin–Ciocalteu reagent (1/6 in deionized water and 600 µL of 20% sodium carbonate after 4 min). After incubation in the dark for 90 min, the absorbance at 750 nm was determined using a Genesys 6 UV spectrophotometer (Thermo Corporation, Waltham, MA, USA). The results were expressed as mg of Gallic acid equivalent per gram of meat in fresh weight.

The determination of flavonoids was performed according to the method of Bag et al. [28], with minor modifications: 0.5 mL of the same extract was mixed with 0.5 mL of methanol (80%), 0.1 mL of aluminum chloride (10%), 0.1 mL of potassium acetate 1 M, and 2.8 mL of deionized water, and then incubated for 30 min at room temperature. Absorbance was measured at 415 nm using a Genesys 6 UV spectrophotometer (Thermo Corporation, USA), and the result was expressed as the mg of quercetin equivalent/g of fresh weight meat.

### 2.8. Lipid and Protein Oxidation

Lipid oxidative status was determined on the 12 fresh and 12 stored samples by cut and by diet following Lynch and Frei’s [29] method with light adaptations as follows [30]. A total of 1.5 g of each muscle was homogenized in 20 mL of extraction buffer (containing 0.15 M of KCl, 0.02 M of EDTA, and 0.30 M of BHT) at 12,000 rpm, 1 min, then were centrifuged at 2000× *g*, 10 min, 4 °C, and 1 mL of the supernatant was taken in a glass tube capped with 1 mL of TBA-TCA (0.07 g TBA, 7 mL of 0.25 M HCl, 7 mL TCA 20%) that was left in boiling water bath for 30 min. Subsequently, they were placed in ice water for 5 min to stop the reaction and cooling for 45 min at room temperature. After that, the pink chromogen was extracted into n-butanol. One ml was extracted and added to 2 mL of n-Butanol and centrifuged at 3000× *g*, 10 min at 4 °C (Thermo Scientific Inc., Waltham, MA, USA). The absorbance in the supernatant was determined at 535 nm in a Genesys-6 spectrophotometer and the concentration of MDA was calculated according to the molar extinction coefficient of malonaldehyde (MDA, 156,000 M^−1^ cm^−1^) and expressed as mg of MDA/kg of meat. A blank sample was prepared for each running. Measurements were run in duplicate. Protein oxidation was determined in the 12 fresh and 12 stored samples of each cut/diet according to the Mercier et al. [31] with light adaptations as follows [30]. A total of 2 mL of the previous homogenate was taken, centrifuged at 3000× *g* for 10 min, 4 °C, then 2 mL of 20 mM DNPH (MERK 3080; 0.048 g of DNPH + 12 mL of 2 M HCl) were added, incubated for 1 h at room temperature, vortexing every 10 min, added 2 mL of 20% TCA and let it rest 15 min, vortexing every 5 min, centrifuged at 2000× *g* for 10 min and separated the pellet. The pellet was washed 3 times in a 1:1 ethanol/ethyl acetate mixture and centrifuged after each wash. After the third wash, the pellet was taken and dissolved with 6 mL of Guanidine (FLUKA 50950) (573.8 g of Guanidine hydrochloride in 1 L of KH_2_PO_4_ prepared from 2.72 g in 1 L of deionized water), and incubated at room temperature for 15 min, vortexing every 5 min. It was centrifuged at 2000× *g* for 10 min and the absorbance of the supernatant measured at 370 nm. The concentration of DNPH was calculated using its molar extinction coefficient of 22,000 M^−1^ cm^−1^, and the results were expressed in nmoles DNPH/mg protein. Also, protein was determined by UV at 280 nm. A blank sample was prepared for each running. Measurements were run in duplicate.

### 2.9. Statistical Analysis

Data are presented as the mean ± SE of 8 pens (3 birds per pen) for each experimental diet for in vivo determinations, and the pen was the experimental unit. For meat quality determinations (pH, color and drip loss), 24 birds were included (as observational unit) for each cut. For analytical determinations of fatty acids and polyphenols, flavonoids and oxidative status, 12 birds for each cut by diet were used. Prior to statistical analyses, the model assumptions of normality and homogeneity of variance were examined by the Shapiro–Wilk and Levene tests, respectively. For growth, feed intake, weight gain, feed conversion rate, carcass weight and carcass yield, a one way-ANOVA was used and as a factor variable, the diet. For the parameters related to cut attributes, one way-ANOVA was applied to analyze the data between inclusion levels of canola meal, and post hoc, the Tukey–Kramer test (*p* < 0.05) was used. For the variables analyzed, taking into account the primary fixed effects, such as diet and process (dose of canola meat and fresh and stored samples), and their interactions, such as polyphenol and flavonoids content and protein and lipid oxidation, GLM ANOVA and post hoc Tukey–Kramer test (*p* < 0.05) were used. NCSS software, v21.0.1–21.0.3 (2021) was used for all analysis of data.

## 3. Results and Discussion

### 3.1. Growth and Feed Intake

During the period from 21 to 42 days, the effect of replacing soybean meal with canola meal (0; 2.5; 5 and 10%) in the finishing diet of meat chickens on the animal’s productive performance was studied. During the study period, all animals showed homogeneous development, and there were no losses due to mortality. The evolution of weekly live weight gain and individual daily and accumulated consumption were registered, from which feed conversion efficiency was calculated (Table 2).

The inclusion of canola meal up to a level of 10% determined live weight evolution values similar to the control diet, normal and even higher than those expected for the Cobb500 line (3272 g; Cobb, 2022). Neither the daily or total feed intake nor the weight gain of the animals was altered in the period considered, where results are similar to the corn–soybean-based control diet. Previous reports by Manyeula et al. [32] using broilers fed CM at 0–17.5% inclusion conclude that rates had comparable feed intake. In addition, our results agree with those found by El-Medany and El-Reffaei [33] in rabbits. These authors explain that the greater weight gain in animals consuming canola meal could be due to the contribution of unsaturated fatty acids (especially linoleic fatty acid) and n-3 that improve lipid metabolism and consequently weight gain. Similar results were found by An et al. [34], who reported a negative effect of canola meal inclusion at 15% of inclusion in the first growing periods of birds (0 to 21 days) but no effect in the growing period (21 to 42 days) even at a 15% level of inclusion when the lysine level is adequate. Negative effects in the growing phase of chicks could be attributed to the high fiber content of canola meal, which could negatively affect weight gain. With age, the development of the digestive tract of the chicken increases and a best tolerance of fiber is attained. In the same sense, Wang et al. [35] determined similar weights in diets that combined soybean and canola meal up to 10% inclusion and in diets with higher inclusion when they were supplemented with an enzyme complex that facilitates the digestion of some non-starch carbohydrates. This result is attributed, according to Ledesma-Mosquera et al. [36] and Cortes et al. [37], to the fact that canola meal has a good amount of some amino acids such as methionine and cysteine, but is low in lysine, so the use of soybean and canola meal together in animal diets could complement their nutritional value. The higher consumption of the diet with 10% inclusion could be an unexpected result compared to the results obtained by Nguyen et al. [38], with growing pigs, who explained this effect by the greater contribution of fiber canola meal compared to soybean meal. In our work, this effect is diluted by the low level of canola meal inclusion given, the low presence of factors that limit the palatability of the food and to the good amino acid profile of canola and the absence of antinutritional factors [39,40].

According to the results calculated for carcass yield, the inclusion of 2, 5 and 5% canola meal in the diet gave similar results to the control group; however, the inclusion of 10% had an opposite effect on this parameter (*p* = 0.05). This result could be due to greater development of the viscera due to the consumption of a diet with a higher fiber content. These data agree with those reported by other authors working on ducks [4], birds [34] and by Nguyen [38] during the finishing stage in pigs.

### 3.2. Meat Quality

**Figure 1:**
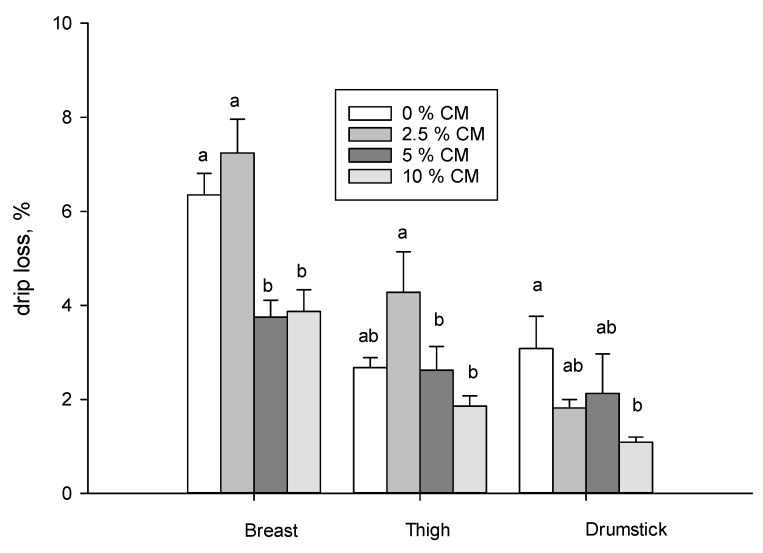
Increasing levels of canola meal in a finishing diet of poultry on the drip loss (%) in breast, thigh and drumstick at 24 h post mortem. a, b means difference significative between levels of CM (0, 2.5, 5 and 10%) for each cut, breast, thigh and drumstick (*n* = 24) by Tukey–Kramer test at *p* < 0.05.

At 24 h post mortem, meat quality parameters were measured: pH, color (Table 3) and drip loss (Figure 1) on the breast, thigh and drumstick cuts.

The effect of the inclusion of canola meal on the pH of the meat was observed, the levels of 5 and 10% of canola meal inclusion determined significantly lower values (5.98 and 5.96 respectively) than the control and the 2.5% level (6.09 and 6.02 respectively) in the breast muscle. In the tight muscle, the pH value was higher at the 10% inclusion level (6.43) than the control (6.54) and the 2.5% and 5% levels (6.50). In the drumstick, no effect was seen on this parameter. However, in all cases, the values remain within the values expected in chicken meat (5, 7 and 6, 1) [35]. The effect of canola meal on the meat pH was largely dependent on the muscle type.

The color of the breast muscle was not influenced by the incorporation of canola meal while the thigh muscle presented darker (*p* < 0.01) colorations compared to the control and the drumstick presented more reddish colorations (*p* < 0.05). Results similar to these were provided by Manyuela et al. [32] who attributed this to the contribution of Fe, Mn, Cu and some pigments from canola, which would stimulate the synthesis of myoglobin and hemoglobin pigments present in greater proportion in oxidative type muscles.

The inclusion of canola meat resulted in a clear reduction in water loss in the breast at the 5% and 10% levels, as well as in the drumstick at the 10% level. The thigh muscle showed more erratic values, with the 5% and 10% inclusion levels being the same as control, while the 2.5% level showed greater water loss (Figure 1).

This parameter is important for industrial processes and for prolonging the useful life of the fresh or stored meat. Meats with a greater capacity to retain water appear tenderer to the consumer [41,42], that is why their benefit is appreciated by both the consumer and the industry. For the three parameters measured, different responses were obtained in each cut due likely to nature, more oxidative or more glycolytic, that impact the biological functions for the animal. Previous studies conducted by our team have demonstrated this [12,43].

### 3.3. Fatty Acids and Lipid Health Indexes in Fresh Cuts

The results of the fatty acid profile study conducted on the fresh three cuts are presented in Table 4.

Concerning breast lipid content, a significant increase (*p* < 0.05) was observed with lower doses of canola meal (2.5%) in poultry diet, while no effect of canola meal on the lipid content for two others cuts was studied (*p* > 0.05; Table 4). The interesting responses were observed related to the fatty acids profile with the inclusion of canola meal in the poultry diet, while modifying the fatty acid profile differently among the various muscles generally favored the concentration of unsaturated fatty acids, monounsaturated and linoleic acid and long-chain fatty acids, particularly in oxidative muscles such as thigh and drumstick cuts. Response is strongly dependent on the muscle type. Lipid metabolism in oxidative and glycolytic muscles is different in spite of the fact that fatty acid in poultry meat reflects fatty acid in diet [12,43]. In this work, saturated fatty acids, such as myristic acid; C14:0, pentadecanoic acid; C15:0, palmitic acid; C16:0 and heptadecanoic acid; C17:0, decreased in the thigh and drumstick, but lignoceric acid; C24:0 only decreased in the breast. This lower content of individuals saturated fatty acids likely provoked a significant decrease in total SFA (∑SFA) in the thigh as a response to canola meal in the diet of poultry (Table 5), but not in the breast or drumstick. Oxidative fibers (type I and type IIa), that are mainly in oxidative muscles in thigh and drumstick cuts are designed for endurance, making them efficient at consuming fat, such as palmitate [44], that could explain the decrease only in the thigh and drumstick (Table 4). While many fatty acids are oxidized in muscle, long saturated fatty acids such as lignoceric acid are primarily associated with the structural lipids, as sphingolipids in myelin and cell membranes and generally show less reliance on immediate oxidative degradation [45]. The breast presents more lignoceric acid (0.69%) than the thigh or drumstick, but 10% of canola meal triggered an increase in C24:0 in the thigh muscle (0.62%) and that is beneficial to cell membrane integrity and as a source of lignoceric acid for human nutrition to prevent heart disease and improve nervous system and skin [45]. However, in this work, canola meal decreases C24:0 content in the breast related to control (Table 4). In the breast, only monounsaturated, particularly oleic acid, increases with all doses related to the control (*p* < 0.01; Table 4). Monounsaturated fatty acids improve the oxidative stability in meat, and this is crucial for the poultry industry [44]. In addition, these fatty acids in poultry meat are considered beneficial to human health, with preventative properties against heart disease and some types of cancer [46,47], and that is why their consumption is recommended. A poultry cut enriched with monounsaturated fatty acid is a high-value animal protein for human health [48]. At inclusion levels of 10%, the n6/n3 ratio in the drumstick and thigh shifted to higher values, due to an increase in C18:2, without any change in n3, which agrees with the results found by Tunnainen et al. [48], who demonstrated this in chickens fed rapeseed oil. However, in the breast, n6/n3 significantly decreases (*p* < 0.05) with 2.5, 5 and 10% of canola meal related to control to more adequate levels considered beneficial for health (<4) [48]. Based on the fatty acid profile, the health indices related to these were determined (Table 5), finding a significant decrease in the thrombogenic and atherogenic indices in the oxidative muscles in the thigh and drumstick cuts, which suggests a favorable effect of the inclusion of canola meal. Beyond the benefits for poultry production and industry, the possibility of producing healthier meat for the consumer is an excellent result of replacing soybeans with canola [49].

Although the physicochemical characteristics of meat are equally crucial for processing and customer acceptance, nutritional values, particularly fatty acids, is crucial and valorize poultry meat with added-value associated with health outcomes [12].

### 3.4. Antioxidants and Oxidative Status in Fresh and Stored Cuts

The polyphenol and flavonoid content of the three muscles fresh and stored was analyzed, and the data are presented in Table 6 and Table 7.

According to these results, polyphenol levels decreased in the breast at 2.5% of canola meal inclusion related to control in fresh and stored cut (*p* < 0.05). No differences were observed in thigh and drumstick cuts (*p* > 0.05) related to control diet. Regarding flavonoids, the breast and drumstick muscles showed the greatest response to canola meal inclusion at 5 and 10% of canola meal (*p* < 0.05). No interactions between diet and process were observed for polyphenols or flavonoids (Table 6 and Table 7). Polyphenols are a major component of the bioactive molecules isolated from plant extracts and particularly phenolic compounds of canola meal show antioxidant activity and present their extract exhibit potential of adipogenesis inhibition [50]. Different types of polyphenols such as lignans, phenolic acids and a variety of flavonoids, and particularly phenolic compounds from canola meal were associated with a decreased risk of chronic vascular disease mortality and in vitro anti adipogenesis effects [51,52]. Concerning the effect in productive animals, polyphenols supplemented improve meat quality in goats [53]. It was shown that polyphenols added in poultry diets significantly enhance meat quality by acting as natural antioxidants, reducing lipid oxidation, and improving muscle color and texture [54]. Also, concerning poultry health, polyphenols improve gut health through the bacteria-derived metabolites of polyphenols that affect the development of the gut and can improve health and productivity of chicken [55]. In our work, total flavonoids from canola meal included in diet at moderate doses increase in the breast and drumstick, and it was probably related to improving the meat quality, particularly the decrease in drip loss. In spite of the fact that polyphenols and flavonoids have low bioavailability, metabolites such as rutin and quercetin could be deposited in meat and an association between polyphenol in diet and total polyphenol in meat was found by Hassan et al. [55]. Since canola contains a great quantity of polyphenols, a large proportion abounds in the residual cake [51,52]. Hussain et al. [51] report that phenolic compounds in canola meal were conjugates and derivatives of hydroxycinnamic acid (sinapic, ferulic, and caffeic acids), and among phenolics, kaempherol as conjugate with sinapic acid being sinapine and trans-sinapic acid the most abundant, as well as major contributors to the antioxidant activity of canola meal extracts [51] depending on the genotype. Clearly, plant genotype, soils, management, environment could affect the polyphenol content as reported in a previous piece of work from our team [56]. Indeed, it is interesting to evaluate the incorporation of individual polyphenols and metabolites of the canola meal used in our work in poultry meat throughout the diet in future research. In our work, only total flavonoids are incorporated in the cuts studied, in the breast and drumstick cuts, and to our knowledge there are no previous reports about this concerning canola. Poultry meat with increased flavonoids is an added value associated with the health attributes. Further research is needed to elucidate this hypothesis.

Lipid and protein oxidation were studied in the three muscles studied, fresh and stored, to evaluate the ability of canola meat in diet to protect the meat during the conservation process (in vacuum, 4–6 °C, 7 days) through the antioxidant compounds as polyphenols content. Data are presented in Table 8 and Table 9.

Lipid oxidation expressed as the mg of MDA/kg of meat remained unchanged in both the fresh and stored cuts studied (*p* < 0.05; Table 8). This response could be associated with the increase in monounsaturated fatty acids in the meat (Table 4) or with the incorporation of flavonoids in the cuts (Table 7), canola meal presents compounds with antioxidant characteristics, mainly phenols and flavonoids [52], an attribute that would protect the increase in PUFAs in the meat of poultry with diets including canola meal without altering its oxidative status in fresh meat or in meat after the display of set days in a vacuum at 4–6 °C. In the breast and thigh, the conservation process appears to have significantly (*p* < 0.05) affected the oxidation of the fatty acids determined by the TBARs procedure in the samples stored in a refrigerated display case, although they remained very low in respect to the maximum acceptable levels (2 mg MDA/kg meat) [57]. Nevertheless, these results were in part expected as lipid oxidation is accelerated by light and storage temperatures (4–6 °C). Therefore, the incorporation of canola meal in diet would have a positive effect on maintaining the stability of the cell wall and probably reducing water loss through dripping observed in this study (Figure 1). When studying the protein oxidation (Table 9) it was affected by the diet, where the 5 and 10% levels presented greater oxidative levels than the 2.5% level in the breast and in the thigh, but in the drumstick, only the level 10% presented greater oxidation (Table 9). The preservation process only affected the oxidative status of the proteins in the drumstick, showing higher oxidation values. This result is noteworthy because protein oxidation is also affected by light and temperature in the display case. Lipid and protein oxidation led to a global evaluation of quality [58]. Oxidation is an important factor in the non-microbial degradation of meat. Oxidized lipids as products of the reaction can readily react with proteins, leading to organoleptic modifications and the loss of nutritional value. Twenty years ago, proteins were considered a target of oxidation [59]. Indeed, protein oxidation could be provoked by secondary products of lipid oxidation [60] or other pro-oxidants that target proteins; this is noteworthy in food rich in proteins such as meat. In this work, since the increase of carbonyls was not along to lipid oxidation (Table 8 and Table 9), and since Gatellier et al. [59] reported that the level of unsaturation of dietary fat was unrelated to the extent of protein carbonylation in pork and beef samples, it is likely that other compounds provoked the aggregation of amino acids in this work [60]. As the pathways of oxidation are more complex and the variety of the oxidative products are greater, protein oxidation and the resulting effects are often less noticeable by consumers [61]. While protein oxidation affects mainly the textural properties of muscle foods, color stability, textural properties or drip loss during the refrigerated storage of meat products [62], the extent of carbonyls formation with the inclusion of canola meal (5 and 10%) observed in this work was not related to a detrimental effect. On the contrary, drip loss largely decreased with 5 and 10% of canola meal in the three cuts studied, being an amazing result of this investigation, especially for the industrial process and gastronomy. Integrating fatty acids composition obtained with an oxidative stability of lipids, a possible hypothesis could be associated with protection by the flavonoids of the canola meal [54]. In addition, it is probable that a different mechanism for each cut was involved, because fatty acids in poultry have a different function in different muscles [12,63]. In the breast, where the main muscle *Pectoralis* is more glycolytic, fatty acids are a source of energy and the increase in ∑MUFA by 10% of canola meal could contribute to regulate metabolism in skeletal muscle that acts as the prior site of glucose storage [64]. The mechanism involved is not clear but it could be directly through physiological mechanisms or indirectly through distinct molecular signal pathways [64]. Numerous flavonoids provide the ability to support bird health while improving the nutritional quality of poultry meat and eggs by changing the profile of fatty acids [65]. Also, flavonoids increased fat deposition in the breast, as reported by Ma et al. [66], and this result was also found in our work with a different profile beneficing monounsaturated fatty acids. This increase is likely associated with oxidative stability provoked by the polyphenols of canola meal. In the thigh and drumstick, with a more oxidative nature, the increase in ∑PUFA, which has a structural function and has a role in transport functions, could also be associated with polyphenols canola and a better oxidative stability. Mainly, n-6 for both muscles and n-3 for the drumstick increase with canola 10% and decrease in saturated fatty acids. In this case, canola, through flavonoids, preserves the PUFA that are the first source for maintenance of the structure and function of the cellules [66]. Taking into account overall effects, flavonoids could be responsible for the oxidative stability related to lipid oxidation observed in this work. While it is unclear how flavonoids could modulate fatty acid composition in the meat and improve the oxidative stability, and further specific research is necessary to elucidate this, the results from previous and present studies suggest that polyphenol content in canola meal has the potential to be used as a dietary ingredient for broilers to improve growth performance and meat quality. However, due to the dual nature of polyphenols since at low concentrations, polyphenols and particularly flavonoids, generally protect proteins by scavenging radicals, it is possible that at high concentrations (>10%), they can act as pro-oxidants by producing excessive ROS [67,68].

## 4. Conclusions

Incorporation of canola meal to partially replace soybean meal up to a level of 10% in the finishing diet of meat chickens does not have a negative effect on final body weight, weight gain or the feed conversion. Canola meal in the poultry diet improved meat quality by reducing water loss, better ultimate pH, color, an enrichment of fatty acids that is noteworthy for human health, good oxidative stability and increased total flavonoids. The meat cuts, such as the breast, thigh and drumstick, due to the nature of main muscles, impacted these responses. Nevertheless, a valuable benefit for industry and consumers with the inclusion of canola in a partial substitution of soybean meal justifies the use of moderate levels of canola meal in poultry diets. However, special focus must be on the protein oxidation of meat in further research in an overall evaluation of canola meal and the impact on the productive performance and the meat quality.

## Figures and Tables

**Table 1:** Ingredients and chemical composition (as feed basis) of the experimental diets containing increasing levels of canola meal offered to chickens from 21 to 42 days of age.

Ingredients (g/kg)	Canola Meal (%)			
	0	2.5	5	10
Yellow corn grain, ground	617.9	605.4	594.9	573.9
Soybean meal, 48%, crude protein	295.0	290.0	274.0	244.0
Canola meal	0.0	25.0	50.0	100.0
Meat and bone meal, 40/45% crude protein	30.0	20.0	20.0	20.0
Monocalcium phosphate, feed grade	5.5	6.0	6.0	6.0
Calcium carbonate, feed grade	6.5	7.5	7.5	7.5
NaCl	2.5	2.5	2.5	2.5
Sunflower oil, high oleic acid	27.0	28.0	29.0	30.0
L-Lysine monochlorhidrate	2.5	2.5	3.0	3.0
DL-Methionine	2.0	2.0	2.0	2.0
Anticoccidial ^1^	0.6	0.6	0.6	0.6
Premix ^2^	10.0	10.0	10.0	10.0
Choline chloride	0.5	0.5	0.5	0.5
Analyzed composition				
Dry matter (%)	88.6	88.6	88.5	88.5
Gross Energy (Mcal/kg)	4.44	4.48	4.68	4.62
Crude Protein (%)	21.3	21.3	21.3	21.3
Lipids (%)	6.02	5.99	6.06	6.12
Crude fiber (%)	4.60	4.80	4.90	5.10
Calcium (%)	0.90	0.90	0.90	0.90
Calculated composition				
Available phosphorus (%)	0.40	0.40	0.40	0.40
Lysine Digestible (%)	1.15	1.16	1.19	1.18
Methionine Digestible (%)	0.49	0.50	0.50	0.51
Arginine Digestible (%)	1.27	1.27	1.27	1.26
Threonine Digestible (%)	0.71	0.72	0.72	0.72

^1^ Monensin sodium. ^2^ The premix (provided the following per kg of premix: 3000.00 KIU vitamin A; 625.00 KIU vitamin D3; 15.63 mg 25 (OH)D3; 20,000.00 mg vitamin E; 800 mg vitamin K3; 800 mg vitamin B1; 2150.00 mg vitamin B2; 1075.00 mg vitamin B6; 4.25 mg de vitamin B12; 16,250.00 mg niacin; 5000.00 mg pantothenic acid; 550.00 mg folic acid; 55,00 mg biotin; 100,000.00 mg choline chloride; 4000.00 mg Cu; 5000.00 mg Fe; 30,000.00 mg Mn; 62.50 mg Co; 312.50 mg Y; 27,500.00 mg Zn; 75.00 mg Se (Rovimix^®^, DSM, Montevideo, Uruguay).

**Table 2:** Effect of increasing doses of canola meal (0, 2.5, 5, 10%) in a finishing diet (21 to 42 days) on animal performance, body weight at slaughter (kg/bird), weight gain (kg/bird), feed intake (kg/bird), feed conversion rate (kg feed/kg weight gain), carcass weight (kg/bird) and carcass yield (%).

Items	Canola Meal (%)				
	0	2.5	5	10	*p*
Final weigh (kg/bird)	3.48 ± 0.07	3.47 ± 0.08	3.46 ± 0.10	3.41 ± 0.09	0.93
Weight gain (kg/bird)	2.19 ± 0.04	2.16 ± 0.05	2.15 ± 0.07	2.12 ± 0.06	0.87
Feed intake (kg/bird)	3.06 ± 0.02	2.93 ± 0.02	3.08 ± 0.02	3.10 ± 0.02	0.74
Feed conversion rate (kg/bird)	1.41 ± 0.03	1.36 ± 0.07	1.44 ± 0.06	1.46 ± 0.06	0.62
Carcass weight (kg/bird)	2.90 ± 0.06	2.89 ± 0.07	2.86 ± 0.08	2.74 ± 0.09	0.44
Carcass yield (%)	83.3 ± 1.5 a	82.3 ± 1.8 ab	82.2 ± 1.9 ab	80.3 ± 2.8 b	0.05

Data are the mean ± SE of *n* = 8 (8 pens × 3 birds) by diet. a, b = Values followed by different lowercase letters indicate significant differences according to the Tukey–Kramer test with *p* < 0.05.

**Table 3:** Effect of the inclusion of increasing doses of canola meal (0, 2.5, 5, 10), on the color (L, a*, b*), pH of breast (*Pectoralis*), thigh (*Ileotibialis*) and drumstick (*Gastrocnemius*).

Items	Cuts	Canola Meal (%)				*p*
		0	2.5	5	10	
**L**	Breast	54.74 ± 1.87	54.70 ± 0.8	54.27 ± 2.33	55.04 ± 2.20	ns
	Thigh	55.77 ± 2.01 a	53.46 ± 1.65 b	51.43 ± 2.46 c	53.55 ± 2.09 b	0.01
	Drumstick	56.44 ± 2.06	55.77 ± 1.52	55.71 ± 2.52	56.22 ± 1.80	ns
a*	Breast	−0.78 ± 0.97	−0.93 ± 0.80	−1.18 ± 0.87	−0.9 ± 0.97	ns
	Thigh	0.704 ± 1.45	1.06 ± 1.07	1.22 ± 1.35	0.69 ± 1.35	ns
	Drumstick	−0.26 ± 0.75 a	−0.28 ± 0.76 a	−0.76 ± 1.03 b	−0.843 ± 0.84 b	0.03
b*	Breast	8.09 ± 1.72	8.14 ± 1.40	7.88 ± 1.15	8.90 ± 2.16	ns
	Thigh	9.00 ± 1.69 a	7.37 ± 1.53 b	6.19 ± 1.91 c	7.68 ± 1.66 b	0.01
	Drumstick	7.14 ± 2.42	7.61 ± 1.88	7.55 ± 1.87	7.34 ± 1.65	ns
**pH**	Breast	6.09 ± 0.09 a	6.02 ± 0.12 a	5.98 ± 0.12 b	5.96 ± 0.12 b	0.01
	Thigh	6.54 ± 0.15 a	6.50 ± 0.13 a	6.50 ± 0.14 a	6.43 ± 0.13 b	0.05
	Drumstick	6.30 ± 0.12	6.31 ± 0.11	6.32 ± 0.16	6.34 ± 0.18	ns

Data are mean ± SE of n = 24. a–c = Values followed by different lowercase letters indicate significant differences between diets by the Tukey–Kramer test (*p* < 0.05). ns = no significant.

**Table 4:** Fatty acids of cuts, breast, drumstick and thigh from birds receiving canola meal (CM) at 0, 2.5, 5 or 10% in a finishing diet.

			Breast				Drumstick					Thigh			
CM, %	0	2.5	5	10	*p*	0	2.5	5	10	*p*	0	2.5	5	10	*p*
Lipids, %	1.72	2.98	2.19	1.93	*	2.45	2.57	2.39	1.88	ns	2.16	2.12	2.43	2.08	ns
Fatty acids, % total fatty acids															
C14:0	0.53	0.57	0.58	0.53	ns	0.54 a	0.53 a	0.53 a	0.44 b	**	0.60 a	0.59 a	0.54 ab	0.44 b	**
C14:1	0.08	0.06	0.06	0.08	ns	0.10 a	0.08 ab	0.08 ab	0.07 b	**	0.08 a	0.07 b	0.09 a	0.08 ab	*
C15:0	0.08	0.10	0.09	0.11	ns	0.09	0.08	0.07	0.07	ns	0.09 b	0.09 b	0.13 a	0.09 b	**
C16:0	24.67	26.41	28.13	25.91	ns	26.86 a	25.52 a	26.14 a	21.69 b	**	26.73 a	27.02 a	26.87 a	22.59 b	*
C16:1	3.97	4.13	4.24	4.26	ns	5.92 a	4.45 b	4.67 b	3.92 c	**	4.74	4.17	4.49	4.06	ns
C17:0	0.27	0.38	0.27	0.42	ns	0.55 a	0.39 ab	0.23 b	0.23 b	**	0.38	0.32	0.28	0.35	ns
C17:1	0.15 a	0.15 a	0.05 b	0.06 b	*	0.19 a	0.08 ab	0.05 b	0.04 b	**	0.11 b	0.15 ab	0.13 ab	0.18 a	ns
C18:0	10.24	9.67	10.90	10.48	ns	8.94	9.88	9.55	8.68	ns	11.44 a	12.09 a	11.79 a	10.42 b	ns
C18:1	45.66 b	55.61 a	52.94 a	54.01 a	**	52.37	54.11	53.42	49.43	ns	50.41	51.74	50.81	46.90	ns
C18:2n6 LNA	8.66 a	1.81 b	1.60 b	3.07 b	*	3.45 b	3.62 b	4.23 b	11.62 a	*	3.98 b	2.60 b	3.57 b	10.59 a	*
C18:3n3 ALA	0.55 a	0.40 b	0.62 a	0.55 a	**	0.51 ab	0.50 b	0.47 b	0.77 a	*	0.51	0.47	0.56	0.53	ns
C20:4n6 ARA	2.42 a	0.06 b	0.08 b	0.07 b	**	0.05 b	0.14 b	0.06 b	0.99 a	*	0.09 b	0.05 b	0.07 b	2.23 a	*
C24:0	0.69 a	0.13 b	0.14 b	0.19 b	*	0.09	0.10	0.09	0.09	ns	0.11 b	0.06 b	0.09 b	0.62 a	**
C22:6n3 DHA	0.34	0.14	0.14	0.30	ns	0.12 b	0.32 a	0.08 b	0.11 b	*	0.27	0.15	0.09	0.37	ns
Others	1.69	0.39	0.16	0.24		0.23	0.22	0.34	1.85		0.46	0.44	0.49	0.56	

Data are the mean of *n* = 12 (SE not show) of each cut, breast, drumstick and thigh for each diet 0, 2.5, 5 and 10% of CM. a–c: means significantly difference between diets by Tukey–Kramer test at <0.05. * *p* < 0.05, ** *p* < 0.01, ns: non significative. LNA = linoleic acid, ALA = alfa linolenic acid, ARA = araquidonic acid, DHA = docosahexaenoic acid.

**Table 5:** Nutritional and lipids health indices of cuts breast, drumstick and thigh of chickens fed diets with increasing levels of canola meal (CM, 0, 2.5, 5 and 10%) in a finishing diet.

	Breast					Drumstick					Thigh				
CM, %	0	2.5	5	10	*p*	0	2.5	5	10	*p*	0	2.5	5	10	*p*
SAT	35.78	37.12	39.97	37.35	ns	36.98	36.39	36.51	31.11	ns	39.23 a	40.11 a	39.60 a	33.87 b	**
ΣMUFA	49.86 b	59.95 a	57.28 a	58.44 a	**	58.57	58.71	58.21	53.45	ns	55.34	56.13	55.52	51.22	ns
ΣPUFA	11.97 a	2.40 b	2.44 b	3.98 b	*	4.13 b	4.58 b	4.84 b	13.50 a	**	4.84 b	3.25 b	4.29 b	13.72 a	*
Σn6	11.08 a	1.87 b	1.67 b	3.14 b	*	3.49 b	3.75 b	4.29 b	12.61 a	*	4.07 b	2.64 b	3.64 b	12.82 a	*
Σn3	0.88 a	0.53 b	0.76 a	0.84 a	*	0.63	0.82	0.54	0.88	ns	0.77	0.61	0.65	0.89	ns
n6/n3	10.11 a	3.43 b	2.40 b	4.58 b	*	5.76 b	4.93 b	7.91 b	12.83 a	**	5.19 b	4.56 b	5.57 ab	11.49 a	*
P/S	0.38 a	0.06 b	0.06 b	0.10 b	*	0.11 b	0.12 b	0.13 b	0.50 a	**	0.12 b	0.08 b	0.10 b	0.44 a	**
AI	1.65	1.73	1.92	1.68	ns	1.74 a	1.65 a	1.69 a	1.33 b	**	1.81 a	1.86 a	1.83 a	1.43 b	**
TI	1.07	1.11	1.22	1.09	ns	1.09 a	1.06 a	1.09 a	0.87 b	**	1.20 a	1.27 a	1.23 a	0.97 b	**
h/H	2.56	2.31	2.08	2.38	ns	2.29 b	2.43 b	2.36 b	3.11 a	**	2.20 b	2.15 b	2.18 b	2.91 a	**

Data are the mean of *n* = 12 (SE not show) of each cut, breast, drumstick and thigh for each diet 0, 2.5, 5 and 10% of CM. a, b: means significantly difference between diets by Tukey–Kramer test at <0.05. * *p* < 0.05, ** *p* < 0.01, ns: non significative. SAT = saturated fatty acids, MUFA = monounsaturated fatty acids, PUFA = polyunsaturated fatty acids, AI = atherogenic index, TI = thrombogenic index, h/H = hypocholelesterolemic index.

**Table 6:** Total polyphenols (TPC, mgGAE/g) content in the breast, thigh and drumstick fresh and stored (7 days, in vacuum, at 4–6 °C), from the birds receiving a finishing diet with canola meal (0, 2.5, 5 and 10%).

Diet	Breast		Thigh		Drumstick	
	Process					
	Fresh	Stored	Fresh	Stored	Fresh	Stored
**0**	1.40 ± 0.07	1.51 ± 0.10	1.60 ± 0.13	1.64 ± 0.12	1.48 ± 0.12	1.61 ± 0.11
**2.5**	1.26 ± 0.17	0.99 ± 0.12	1.48 ± 0.14	1.23 ± 0.13	1.46 ± 0.12	1.22 ± 0.12
**5**	1.10 ± 0.08	1.37 ± 0.14	1.41 ± 0.12	1.59 ± 0.14	1.44 ± 0.11	1.59 ± 0.13
**10**	1.26 ± 0.10	1.13 ± 0.10	1.60 ± 0.12 0.1152262	1.37 ± 0.14	1.74 ± 0.11	1.37 ± 0.12
Main effects						
Diet Process DxP	*p* < 0.02; 0 > 2.5 ns ns		ns ns ns		ns ns ns	

Data are mean ± SE of *n* = 12 for each cut, breast, thigh and drumstick, fresh or stored. Main effects was analyzed by ANOVA GLM with fixed effects diet and process, interactions DxP, and post hoc Tukey–Kramer test (*p* < 0.05), ns = not significant.

**Table 7:** Total flavonoids content (TFC, µgQE/g) content in the breast, thigh and drumstick fresh and stored (7 days, in vacuum, at 4–6 °C), from the birds receiving a finishing diet with canola meal (0, 2.5, 5 and 10%).

Diet	Breast		Thigh		Drumstick	
	Process					
	Fresh	Stored	Fresh	Stored	Fresh	Stored
**0**	259 ± 22 b	272 ± 20 a	261 ± 16 a	278 ± 17 a	246 ± 15 b	253 ± 16 b
**2.5**	239 ± 22 b	276 ± 18 a	197 ± 16 b	287 ± 16 a	200 ± 17 c	290 ± 15 a
**5**	332 ± 17 a	209 ± 18 b	268 ± 15 a	251 ± 17 b	286 ± 14 a	204 ± 15 c
**10**	281 ± 19 ab	276 ± 19 a	244 ± 17 ab	269 ± 17 ab	255 ± 16 a	294 ± 15 a
Main effects						
Diet Process DxP	ns ns *p* < 0.01		ns *p* < 0.02; S > F *p* < 0.01		ns ns *p* < 0.01	

Data are mean ± SE of *n* = 12 for each cut, breast, thigh and drumstick, fresh (F) or stored (S). Main effects was analyzed by ANOVA GLM with fixed effects diet and process, interactions DxP, and Tukey–Kramer test (*p* < 0.05). a–c: means significant difference between diets for fresh or stored cuts by ANOVA one way (*p* < 0.05), ns = not significant.

**Table 8:** Lipid oxidation expressed as TBARS (mg MDA/kg meat) in the breast, thigh and drumstick fresh and stored (7 days, in vacuum, at 4–6 °C), from the birds receiving a finishing diet with canola meal (0, 2.5, 5 and 10%).

Diet	Breast		Thigh		Drumstick	
	Process					
	Fresh	Stored	Fresh	Stored	Fresh	Stored
**0**	0.31 ± 0.01	0.44 ± 0.04	0.44 ± 0.05	0.53 ± 0.07	0.33 ± 0.03	0.43 ± 0.05
**2.5**	0.35 ± 0.03	0.42 ± 0.05	0.42 ± 0.04	0.48 ± 0.04	0.48 ± 0.05	0.46 ± 0.05
**5**	0.44 ± 0.05	0.47 ± 0.03	0.46 ± 0.04	0.52 ± 0.05	0.47 ± 0.03	0.46 ± 0.03
**10**	0.37 ± 0.02	0.47 ± 0.05	0.41 ± 0.03	0.51 ± 0.03	0.43 ± 0.04	0.49 ± 0.05
**Main effects**						
Diet Process DxP	ns *p* < 0.05; F, 0.37 < S, 0.45 ns		ns *p* < 0.05; F, 0.43 < S, 0.51 ns		ns ns ns	

Data are mean ± SE of n = 12 for each cut, breast, thigh and drumstick, fresh (F) or stored (S). Main effects was analyzed by ANOVA GLM with fixed effects diet and process, interactions DxP, and Tukey–Kramer test (*p* < 0.05), ns = not significant.

**Table 9:** Protein oxidation expressed as carbonyls (nM DNPH/mg protein) in the breast, thigh and drumstick fresh and stored (7 days, in vacuum at 4–6 °C), from the birds receiving a finishing diet with canola meal (0, 2.5, 5 and 10%).

Diet	Breast		Thigh		Drumstick	
	Process					
	Fresh	Stored	Fresh	Stored	Fresh	Stored
**0**	0.84 ± 0.11	1.07 ± 0.14	0.78 ± 0.05	1.04 ± 0.13	0.60 ± 0.11	0.98 ± 0.16
**2.5**	0.79 ± 0.11	0.89 ± 0.16	0.48 ± 0.08	0.73 ± 0.15	0.60 ± 0.09	0.79 ± 0.15
**5**	1.03 ± 0.06	1.18 ± 0.17	0.98 ± 0.13	1.06 ± 0.12	0.86 ± 0.09	1.03 ± 0.11
**10**	1.14 ± 0.06	1.21 ± 0.13	1.18 ± 0.07	1.08 ± 0.16	1.01 ± 0.09	1.06 ± 0.12
**Main effects**						
Diet Process DxP	*p* < 0.05; 2.5% < 5, 10% ns ns		*p* < 0.05; 2.5% < 5, 10% ns ns		*p* < 0.05; 2.5% < 10% *p* < 0.05; F < S ns	

Data are mean ± SE of n = 12 for each cut, breast, thigh, drumstick, fresh (F) or stored (S). Main effects was analyzed by ANOVA GLM with fixed effects diet and process, interactions DxP, and Tukey–Kramer test (*p* < 0.05), ns = not significant.

## Data Availability

The original contributions presented in this study are included in the article. Further inquiries can be directed to the corresponding authors.
